# Donor–Recipient Story in Allogeneic Hematopoietic Stem Cell Transplantation

**DOI:** 10.3390/curroncol28010067

**Published:** 2021-01-24

**Authors:** Elena Kum, Gabriele Jagelaviciute, Edward Li, Kenneth Williams, Santhosh Thyagu, Warren Fingrut

**Affiliations:** 1 Stem Cell Club, Toronto, ON M2H 3L2, Canada; elenakum@rogers.com (E.K.); gjagelav@uwo.ca (G.J.); edward.li.wei@gmail.com (E.L.); kenneth.williams@mail.utoronto.ca (K.W.); 2 Faculty of Health Sciences, McMaster University, Hamilton, ON L8S 4L8, Canada; 3 Faculty of Medicine, Queen’s University, Kingston, ON K7L 3N6, Canada; 4 Faculty of Medicine, University of Toronto, Toronto, ON M5S 1A1, Canada; Santhosh.Thyagu@uhn.ca; 5 Division of Medical Oncology and Hematology, Princess Margaret Cancer Centre, Toronto, ON M5G 2M9, Canada; 6 Faculty of Medicine, University of British Columbia, Vancouver, BC V6T 1Z3, Canada; 7 Adult Bone Marrow Transplantation Service, Department of Medicine, Memorial Sloan Kettering Cancer Center, 1275 York Avenue, Box 298, New York, NY 10065, USA

**Keywords:** stem cell transplantation, bone marrow transplantation, donation, unrelated donors

## Abstract

Patients with a variety of blood, immune, and metabolic disorders may require an allogeneic hematopoietic stem cell transplant as part of their treatment. However, over 70% of these patients do not have a matched sibling donor and require an alternative donor, such as a matched unrelated donor. We present a multi-part story of a Canadian stem cell recipient who underwent transplantation for treatment of refractory chronic myelogenous leukemia, and the matched unrelated donor who saved his life. The story segments feature excerpts from interviews with the donor and the recipient, along with representative images of both storytellers. The excerpts were optimized for publication on social media and were arranged to build a story arc that parallels the journey of the donor and recipient together. This donor-recipient story may serve as a resource to help raise awareness about stem cell donation and to encourage eligible individuals to register as donors. The story is one of several developed by Why We Swab, a library of stories in stem cell donation in Canada (Facebook, Twitter, and Instagram; @WhyWeSwab) to support the recruitment of committed unrelated donors.

Patients with a variety of blood, immune, and metabolic disorders may require an allogeneic hematopoietic stem cell transplant as part of their treatment. However, over 70% of these patients do not have a matched sibling donor and require an alternative donor, such as a matched unrelated donor. We present a multi-part story of a Canadian stem cell recipient who underwent transplantation for treatment of refractory chronic myelogenous leukemia, and the matched unrelated donor who saved his life (Figure 1). The story segments feature excerpts from interviews with the donor and the recipient, along with representative images of both storytellers. The excerpts were optimized for publication on social media and were arranged to build a story arc that parallels the journey of the donor and recipient together.

The story begins by capturing the accounts of the recipient in need of an unrelated stem cell donor (part 1) and his donor joining the Canadian stem cell donor registry (part 2). The donor then recounts giving stem cells from his blood (part 3). The story continues with the recipient’s initial contact with his donor (part 4), and their first in-person meeting (part 5), highlighting the development of their emotional connection. The story concludes with the recipient’s reflection on how his donor has impacted his life (part 6), with a photo of the recipient giving a speech at the donor’s wedding.

Sharing stories is an effective tool for promoting health education and awareness [1]. Stories can elicit a strong emotional connection with the storyteller, helping people to empathize with the experience of others [2]. They can act as a source of information and advice, and they can give storytellers and listeners feelings of recognition, appreciation, and understanding [2]. They can also transform medical knowledge into narratives that are more universally understood [3]. We share the journey of a stem cell recipient and his donor not only to honor the experiences of both storytellers, but also to raise awareness about stem cell donation and to provide a resource to patients, donors, caregivers, and transplant staff.

To our knowledge, this is the first donor–recipient story in the world which is told in this format. Online communication technologies allow patients to express their experiences of illness to a larger, real-time audience and to facilitate sharing and comments. Some cancer patients maintain online blogs, which allow them to publish and disseminate long-form narratives about their experiences with disease and treatment, often from diagnosis through life as a survivor [4]. We arranged this donor–recipient story into short segments suitable for publication across social media channels (including Facebook, Instagram, and Twitter) in order to engage a younger demographic that represents ideal potential donors [5]. In addition, this format supports the dissemination of this story to relevant public audiences.

For patients and caregivers, this story can help to inform or give a sense of encouragement about finding a match or going through the transplant process. The emotions and challenges that are captured in this story may help to reassure and provide hope to patients and caregivers that they are not alone in their experience. For healthcare providers, this story can give insight into the experience of stem cell donors and recipients. It can help them to appreciate the complex journey of those impacted by stem cell donation, including how that journey is experienced, understood, and represented. This can support the practice of narrative medicine, which involves mutual learning, understanding, and empathy between patients and health care providers as a means to improve patient care [6]. For past donors, this story may serve as a reminder of their life-saving gift of the donation of stem cells. Sharing this story can be a way to honor and express appreciation for all donors who have impacted a recipient and their loved ones. For registered donors, this story may help to reaffirm their commitment as donors, and remind them of the hope they provide should they match with a patient in need. Lastly, in addition to serving as an introduction to stem cell donation for the general public, this story conveys how donation can impact patients, donors, caregivers, and families. This story can be shared to raise awareness about stem cell donation and to encourage eligible individuals to register as donors; in Canada, this is done either online (via Canadian Blood Services or Héma-Québec) or at a stem cell drive, where registrants provide their consent and a tissue sample for human leukocyte antigen (HLA)-typing [7]. Individuals in Canada are eligible to register as stem cell donors if they are between the ages of 17 and 35, in good general health, willing to help anyone in need, and have Canadian healthcare coverage. We hope that in sharing this story, we can provide hope for patients searching for a match, and inspire others to register as stem cell donors.

**Figure 1:**
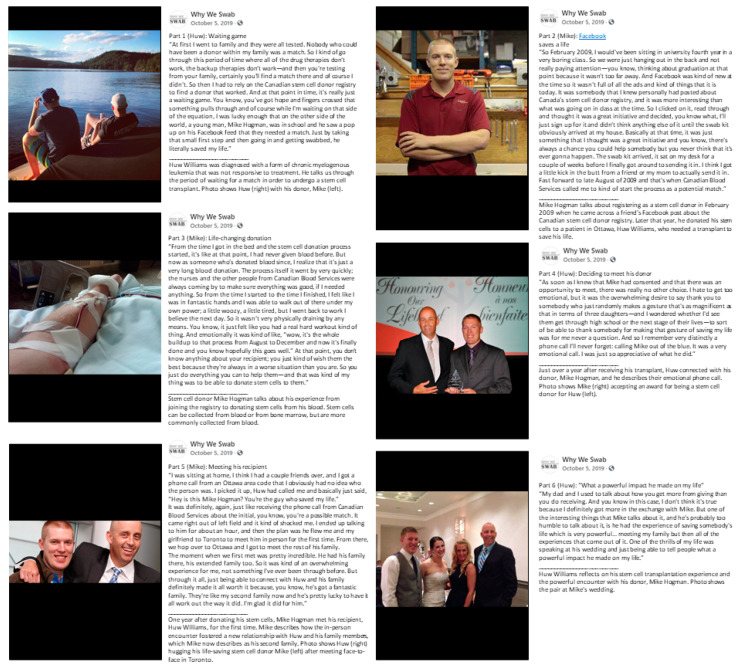
This donor–recipient story is one of several stories regarding stem cell donation by Why We Swab, a library of Canadian stories in stem cell donation (Facebook, Instagram, and Twitter; @WhyWeSwab) [8]. This multi-part story was shared in a series of posts featuring excerpts from interviews with the donor and the recipient, paired with the storytellers’ photos.

## Data Availability

Data sharing not applicable.
